# Effect of electronic health (eHealth) on quality of life in women with breast cancer: A systematic review and meta‐analysis of randomized controlled trials

**DOI:** 10.1002/cam4.6094

**Published:** 2023-05-18

**Authors:** Tianmeng Wen, Chongxiang Chen, Wenhui Ren, Shangying Hu, Xuelian Zhao, Fanghui Zhao, Qinyu Zhao

**Affiliations:** ^1^ Department of Epidemiology National Cancer Center/National Clinical Research Center for Cancer/Cancer Hospital Chinese Academy of Medical Sciences and Peking Union Medical College Beijing China; ^2^ Department of ICU, State Key Laboratory of Oncology in South China, Collaborative Innovation Center for Cancer Medicine Sun Yat‐sen University Cancer Center Guangzhou China; ^3^ Guangzhou Development District Hospital Guangzhou China

**Keywords:** breast cancer, eHealth, meta‐analysis, quality of life

## Abstract

**Background:**

Women with breast cancer and improved survival face some specific quality of life (QOL) issues. Electronic health (eHealth) is a useful tool aiming to enhance health services. However, evidence remains controversial about the effect of eHealth on QOL in women with breast cancer. Another unstudied factor is the effect on specific QOL functional domains. Therefore, we undertook a meta‐analysis about whether eHealth could improve the overall and specific functional domains of QOL in women with breast cancer.

**Methods:**

PubMed, Cochrane Library, EMBASE, and Web of Science were searched to identify appropriate randomized clinical trials from database inception to March 23, 2022. The standard mean difference (SMD) served as the effect size and the DerSimonian–Laird random effects model was constructed for meta‐analysis. Subgroup analyses were conducted according to different participant, intervention, and assessment scale characteristics.

**Results:**

We initially identified 1954 articles excluding duplicates and ultimately included 13 of them involving 1448 patients. The meta‐analysis revealed that the eHealth group had significantly higher QOL (SMD 0.27, 95% confidence interval [95% CI] 0.13–0.40, *p* < 0.0001) than the usual care group. Additionally, although not statistically significant, eHealth tended to improve the physical (SMD 2.91, 95% CI −1.18 to 6.99, *p* = 0.16), cognitive (0.20 [−0.04, 0.43], *p* = 0.10), social (0.24 [−0.00, 0.49], *p* = 0.05), role (0.11 [0.10, 0.32], *p* = 0.32), and emotional (0.18 [0.08, 0.44], *p* = 0.18) domains of QOL. Overall, consistent benefits were observed in both the subgroup and pooled estimates.

**Conclusions:**

eHealth is superior to usual care in women with breast cancer for improved QOL. Implications for clinical practice should be discussed based on subgroup analysis results. Further confirmation is needed for the effect of different eHealth patterns on specific domains of QOL, which would help improve specific health issues of the target population.

## 1 INTRODUCTION

Breast cancer is the most common malignancy among women worldwide.^1^ The global mortality‐to‐incidence rate was about 36% in 2000 and decreased to 28% in 2020, which means women with breast cancer (including patients and survivors [defined as no cancer recurrence]) live longer than before due to the implementation of mammography‐based screening and improved treatments.^1^, ^2^

Quality of life (QOL) is of particular importance to breast cancer among women with a longer survival time. However, they face some specific QOL issues that make their lives different from those of healthy people, including intimacy issues,^4^ loss of womanhood,^5^ and body image distortion, along with the disruptive effects of treatments.^6^ Therefore, we need to take the QOL of women with breast cancer into consideration, instead of focusing only on their survival time.

Electronic health (eHealth) is a useful tool aiming to enhance health services and collect patient information through the internet and related technologies (e.g., websites, software, digital gaming, etc.), and helps to tackle barriers including distance, time, cost, and lack of health providers.^3^ For example, mobile applications (apps) were found to help solve certain health problems without increasing the risk of exposure to the 2019 novel coronavirus disease (COVID‐19) during the pandemic.^4^ In addition, Mobile apps are well‐accepted by women with breast cancer.^5^ Since the evidence remains controversial about the effect of eHealth on QOL in women with breast cancer,^6^, ^7^, ^8^, ^9^ it is important to conduct a systematic review and meta‐analysis to conclude. Another unstudied factor is the effect of eHealth on specific functional domains of QOL, including physical, cognitive, social, role, and emotional domains.^10^, ^11^, ^12^, ^13^, ^14^ Hence, our study aimed to conduct a meta‐analysis to explore the effects of eHealth interventions on the overall and five functional domains of QOL.

## 2 MATERIALS AND METHODS

### 2.1 Search strategy

Two investigators independently searched for articles in four databases (PubMed, Cochrane Library, EMBASE, and Web of Science). Medical subject headings and other terms were used in the searches, including “telemedicine,” “eHealth,” “mobile health,” “mHealth,” “telehealth,” “telemonitor,” “telemanagement,” “telecare,” “telerehabilitation,” “breast neoplasms,” “breast carcinoma,” “breast cancer,” and “breast tumor.” The search terminal date was March 23, 2022.

### 2.2 Inclusion and exclusion criteria

According to the PICOs (population, intervention, comparison, and outcome), the following inclusion criteria were established: (1) population: women currently or previously diagnosed with breast cancers, regardless of whether completion of medical treatment (e.g., surgery, adjuvant chemotherapy, and radiotherapy); (2) intervention and comparison: eHealth intervention and usual care. eHealth intervention should be healthcare delivered through the internet and related technologies (e.g., websites, software, etc.). Usual care referred to basic healthcare services, including traditional health education (e.g., paper‐based instruments) and routine nursing care, etc.; (3) outcome: primary outcome being health‐related QOL. The assessment instruments and follow‐up duration were not restricted; (4) study design: randomized clinical trials (RCTs); (5) studies being published in English.

The exclusion criteria were (1) studies including other cancers, (2) significant baseline differences between groups, (3) a review, retrospective study, or case report, (4) not divided into eHealth group and control group, and (5) insufficient data in the article.

### 2.3 Data extraction

Two authors independently reviewed the identified articles. The following baseline and study characteristics were extracted from each included publication: publication information, participant characteristics, intervention method and duration, and QOL assessment scales (Tables 1 and 2).

**TABLE 1:** Characteristics of the studies included in the meta‐analysis.

Study	Country	Income level^a^	Jadad scale^b^	Center	Follow up	Sample (eHealth vs. control)	Age (eHealth vs. control)	Participant	QOL scales	Intervention
									Anxiety scales	
									Depression scales	
Admiraal (2017)	Netherlands	High‐income	2 + 2 + 0 + 1 = 5	Multi‐center	12 weeks	62 vs. 63	53.1 ± 9.8 vs. 53.2 ± 8.5	Patients who recently completed surgery combined with adjuvant chemotherapy within the past 6 months	EORTC QLQ‐C30	Non‐mobile: web‐based tailored psycho‐educational program; Communication
David (2011)	Germany	High‐income	1 + 1 + 0 + 1 = 3	Single‐center	2 months	31 vs. 34	48.2 ± 9.2 vs. 45.9 ± 7.8	Patients with the TNM staging system ranging from T1 and G1 to T4 or N1 or M1	EORTC QLQ‐C30	Non‐mobile: psychosocial counseling by web‐based e‐mail; Communication
									BSI	
									BSI	
Freeman (2015)	USA	High‐income	1 + 1 + 0 + 1 = 3	Multi‐center	3 months	23 vs. 47	55.57 ± 9.88 vs. 55.28 ± 7.90	At least 6 weeks after completing their major cancer treatments	SF‐36	Non‐mobile: video conferencing software; Communication
Galiano‐Castillo (2016)	Spain	High‐income	2 + 2 + 0 + 1 = 5	Single‐center	6 months	39 vs. 37	47.4 ± 9.6 vs. 49.2 ± 7.9	I–IIIA, survivors had completed adjuvant therapy	EORTC QLQ‐C30	Non‐mobile: Internet‐Based Exercise Intervention; Non‐communication
Hou (2020)	China	Upper‐middle‐income	2 + 1 + 0 + 1 = 4	Multi‐center	3 months	48 vs. 52	Range 20–64	0–III, patients	EORTC QLQ‐BR23	Mobile: Self‐Management Support mHealth App; Communication
Kim (2018)	Korea	High‐income	2 + 1 + 0 + 1 = 4	Single‐center	3 weeks	36 vs. 40	49.8 ± 13.5 vs. 52.1 ± 13.2	IV, patients, a combination of at least third‐line palliative chemotherapy	WHOQOL‐BREF	Mobile: mobile game developed to improve self‐management; Non‐communication
									STAI	
									BDI	
Owen (2005)	UK	High‐income	2 + 1 + 0 + 0 = 3	Single‐center	12 weeks	26 vs. 27	52.5 ± 8.6 vs. 51.3 ± 10.5	0–III, survivors	FACT‐B	Non‐mobile: Self‐Guided Internet Coping Group; Communication
Ryhanen (2012)	Finland	High‐income	2 + 1 + 1 + 1 = 5	Single‐center	1 year	47 vs. 43	54.4 vs. 55.7	0–IV, patients come to have surgery	Quality of Life instrument– Breast Cancer Patient Version	Non‐mobile: Empowering Internet‐based Breast Cancer Patient Pathway program; Non‐communication
									STAI	
Uhm (2016)	Korea	High‐income	2 + 1 + 0 + 1 = 4	Multi‐center	12 weeks	167 vs. 172	49.3 ± 8.0 vs. 51.3 ± 10.7	0–IV, early breast cancer survivors	EORTC QLQ‐C30	Mobile: mobile health with a pedometer; Non‐communication
Visser (2018)	Netherlands	High‐income	2 + 1 + 0 + 1 = 4	Single‐center	6 months	46 vs. 41	55.8 ± 8.3 vs. 57.9 ± 8.8	Patients with primary treatment at most 5 years ago	EORTC QLQ‐C30	Mobile: internet (my medical consultations) online apps; Communication
Van‐den‐ Berg (2015)	Netherlands	High‐income	2 + 2 + 0 + 1 = 5	Multi‐center	4 months	70 vs. 80	51.44 ± 8.30 vs. 50.18 ± 9.15	Early breast cancer survivors	EORTC QLQ‐C30	Non‐mobile: web‐based self‐management intervention; Non‐communication
Zhou (2020)	China	Upper‐middle‐income	2 + 1 + 2 + 0 = 5	Single‐center	6 months	55 vs. 48	49.84 ± 8.85 vs. 49.98 ± 9.84	I–III, newly diagnosed with breast cancer, surgery with adjuvant therapy	FACT‐B	Mobile: WeChat‐based multimodal nursing program; Communication
Zhu (2018)	China	Upper‐middle‐income	2 + 2 + 0 + 1 = 5	Multi‐center	6 months	57 vs. 57	46.2 ± 8.5 vs. 48.2 ± 8.1	0–IV, patients, during chemotherapy	FACT‐B	Mobile: Mobile Breast Cancer e‐Support Program; Communication
									HADS	
									HADS	

Abbreviations: apps, applications; BDI, Beck Depression Inventory; BSI, Brief Symptom Inventory; eHealth, electronic health; EORTC QLQ‐C30, The European Organization for Research and Treatment of Cancer Quality of Life Questionnaire‐Core30; EORTC QLQ‐BR23, The European Organization for Research and Treatment of Cancer‐Breast Cancer‐Specific Quality‐of‐Life Questionnaire; FACT‐B, Functional Assessment of Cancer Therapy‐Breast Cancer Form; HADS, Hospital Anxiety and Depression Scale; QOL, quality of life; RCT, randomized controlled trial; SF‐36, Medical Outcomes Study 36‐item short‐form survey; STAI, State–Trait Anxiety Inventory; WHOQOL‐BREF, World Health Organization Quality of Life‐Bref Scale.

^a^ According to the World Bank Country and Lending Groups (https://datahelpdesk.worldbank.org/knowledgebase/articles/906519‐world‐bank‐country‐and‐lending‐groups).

^b^ Randomization + Concealment of allocation + Double blinding + Withdrawals and dropouts.

**TABLE 2:** EORTC QLQ‐C30 functional scales.

Study	Physical (eHealth vs. control)	Social (eHealth vs. control)	Emotional (eHealth vs. control)	Cognitive (eHealth vs. control)	Role (eHealth vs. control)
Admiraal (2017)	6.42 ± 15.33 vs. 9.21 ± 13.52	11.02 ± 20.46 vs. 8.47 ± 25.38	2.29 ± 19.06 vs. 1.41 ± 17.26	3.97 ± 20.02 vs. 5.03 ± 14.86	9.68 ± 25.53 vs. 11.11 ± 25.92
David (2011)	‐	52.15 ± 34.36 vs. 50.49 ± 31.11	44.62 ± 31.37 vs. 42.65 ± 26.41	59.14 ± 33.57 vs. 56.86 ± 31.28	53.76 ± 35.15 vs. 52.94 ± 30.00
Galiano‐Castillo (2016)	85.13 ± 12.93 (80.94–89.32) vs. 74.59 ± 19.68 (68.03–81.15)	84.61 ± 21.42 (77.67–91.56) vs. 65.76 ± 30.16 (55.71–75.82)	77.5 ± 24.04 (69.77–85.36) vs. 58.33 ± 32.03 (47.65–69.01)	78.20 ± 21.68 (71.18–85.23) vs. 60.81 ± 30.48 (50.65–70.97)	82.05 ± 24.60 (74.08–90.02) vs. 68.92 ± 25.51 (60.41–77.42)
Uhm (2016)	83.4 ± 11.8 vs. 81.3 ± 13.3	81.9 ± 21.8 vs. 78.9 ± 24.7	78.7 ± 17.0 vs. 75.9 ± 20.2	79.8 ± 16.3 vs. 78.5 ± 20.0	82.6 ± 18.6 vs. 83.0 ± 19.1

Abbreviations: EORTC QLQ‐C30, The European Organization for Research and Treatment of Cancer Quality of Life Questionnaire‐Core30; eHealth, electronic health.

### 2.4 Risk of bias assessment

The risk of bias in the clinical trials included in our meta‐analysis was assessed according to the recommendations of the Cochrane Handbook of Systematic Reviews of Interventions (http://handbook.cochrane.org.), including the following domains: selection bias (random sequence generation and concealment of allocation), performance bias (blinding of participants and personnel), detection bias (blinding of outcome assessment), attrition bias (incomplete outcome data), and reporting bias (selective outcome reporting).

### 2.5 Quality assessment

The methodological quality of studies in this meta‐analysis was evaluated by the Jadad Scale, which scores from 0 to 7 (0–3: low quality; 4–7: high quality). With this tool, each study was assessed in four separate categories: randomization, concealment of allocation, double blinding, and withdrawals and dropouts.^15^ The Jadad Scale scores of the included studies ranged from 3 to 5 (Table 1).

### 2.6 Statistical analysis

Primary outcomes were measured by the difference in QOL (including overall and five functional domains of QOL) between eHealth and control groups. The overall QOL was measured by the total score of each scale included in our studies shown in Table 1; the functional outcomes were measured by the European Organization for Research and Treatment of Cancer Quality of Life Questionnaire‐Core 30 (EORTC QLQ‐C30), which includes scales measuring physical, role, emotional, cognitive, and social domains. Higher scores of overall QOL or functional domains represent better QOL or functioning. Anxiety, as measured by the State–Trait Anxiety Inventory (STAI), the Hospital Anxiety and Depression Scale (HADS), and the Brief Symptom Inventory (BSI), was a secondary outcome of our study; depression was another secondary outcome measured by the Beck Depression Inventory, the BSI, and the HADS. For scales measuring anxiety and depression, higher scores mean worse issues. We used the standard mean difference (SMD) and a corresponding 95% confidence interval (95% CI) between the eHealth and control groups as the effect size. Given the clinical and methodological quality heterogeneity across trials, the DerSimonian–Laird random effects model was constructed for pooled estimates.

Between‐study heterogeneity was quantified by the *I* ^2^ statistic and interpreted qualitatively as low (25%–50%), moderate (50%–75%), and high (75%–100%).^16^ To identify the sources of heterogeneity, the subgroup, sensitivity, and meta‐regression analyses were performed. The meta‐regression analysis also investigated if any covariate moderate treatment effect sizes. Covariates for subgroup and meta‐regression analyses included health status (patients vs. survivors), medical treatment (undergoing vs. not undergoing), income level (high‐income vs. upper‐middle‐income countries), whether communication with health providers (yes vs. no), whether mobile‐based (yes vs. no), follow‐up period (≤3 months vs. 3–12 months), QOL assessment scales (breast cancer‐specific vs. cancer‐specific vs. general), study quality (high [Jadad scale score >3] vs. low [Jadad scale score ≤3]) and the publication year (within last 10 years vs. earlier years). Sensitivity analyses were conducted by omitting each study in turn. Publication bias was assessed through funnel plots. Meta‐regression analysis was performed by Stata/SE 16.0 (StataCorp LLC) while other analyses were by Review Manager 5.4 (The Cochrane Collaboration).

## 3 RESULTS

### 3.1 Study characteristics

**FIGURE 1:**
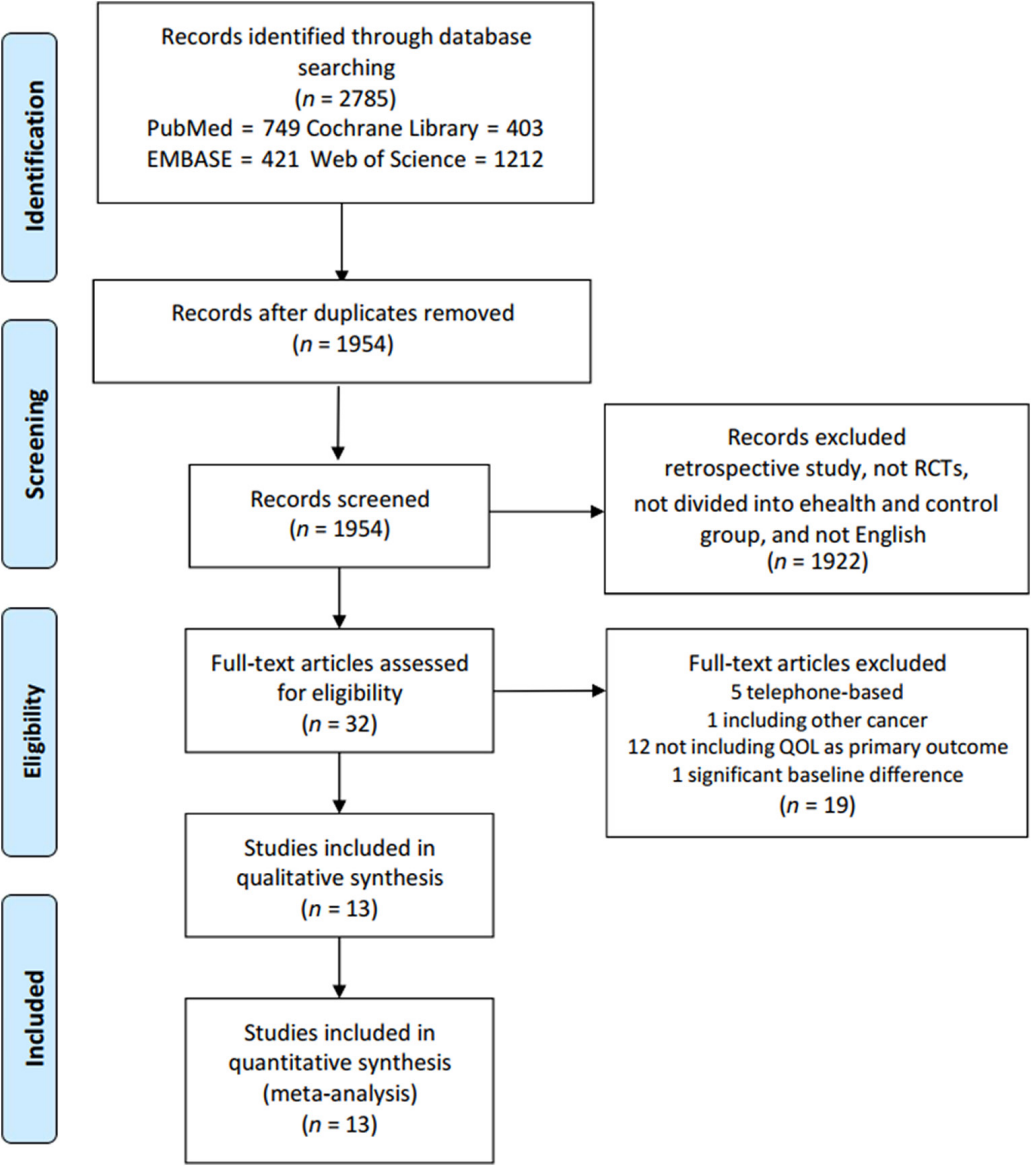
Flow diagram of choosing the appropriate articles. QOL, quality of life; RCTs, randomized clinical trials.

**FIGURE 2:**
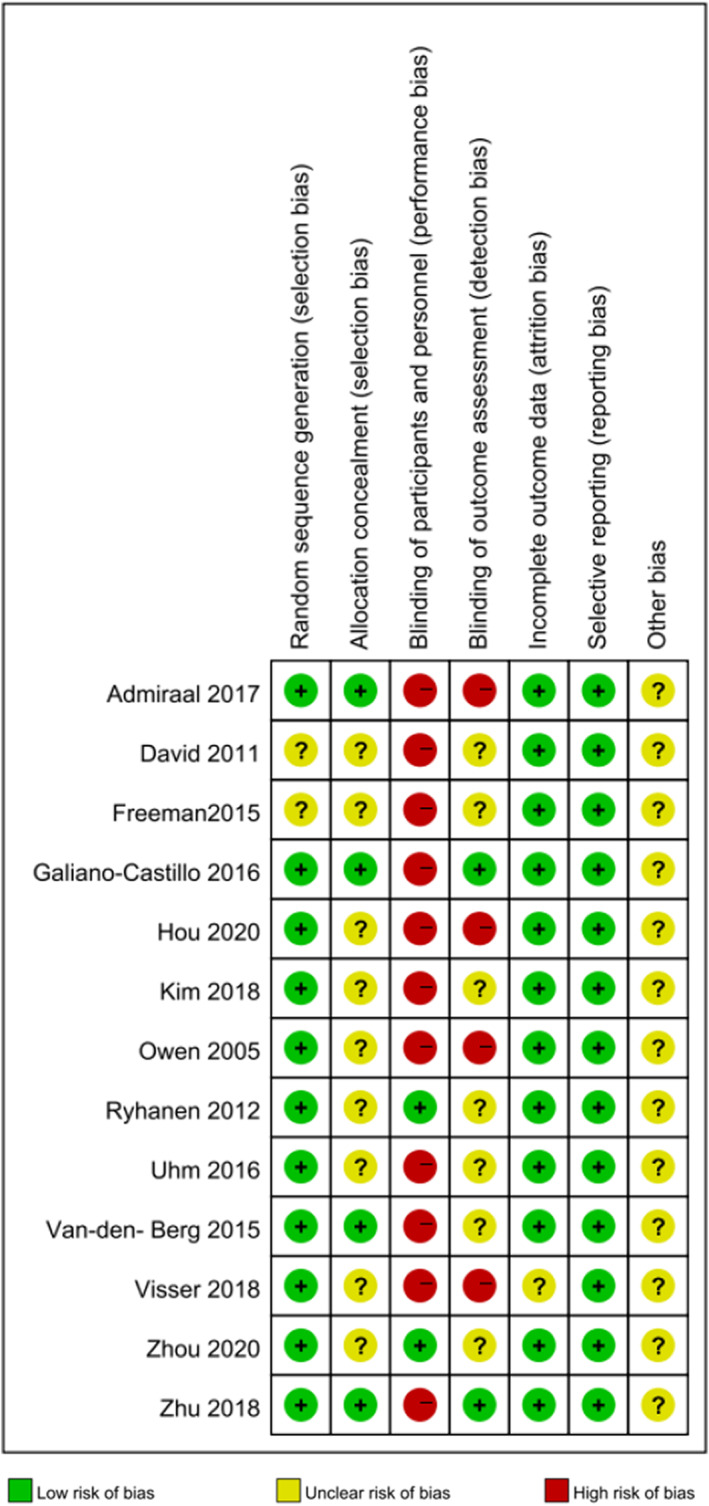
Risk of bias summary.

We initially identified 1954 articles excluding duplicates, and then 1922 of these articles were excluded by reading the title and abstract. Ultimately, 13 articles involving 1448 patients (707 in the eHealth group and 741 in the control group) were included in the meta‐analysis after reading the entire articles^6^, ^7^, ^8^, ^9^, ^11^, ^17^, ^18^, ^19^, ^20^, ^21^, ^22^, ^23^, ^24^ (Figure 1). The basic characteristics of the included trials and the demographic characteristics of their participants are shown in Table 1. Included studies were randomized trials published from 2005 to 2020. Ten studies were conducted in high‐income countries: Netherlands,^17^, ^18^, ^24^ Germany,^19^ the United States of America,^20^ Spain,^9^ Finland,^8^ Korea,^21^, ^23^ the United Kingdom^22^; and three in upper‐middle‐income country: China.^6^, ^7^, ^11^ Regarding study population and intervention, nine studies included breast cancer patients,^6^, ^7^, ^8^, ^11^, ^17^, ^19^, ^20^, ^21^, ^24^ and four included breast cancer survivors^18^, ^22^, ^23^; seven studies involved non‐mobile‐based eHealth platforms (software,^20^ websites,^17^, ^18^, ^19^, ^22^ pathway programs,^8^ or a combination of the former two^9^) and six included mobile‐based platforms (apps).^6^, ^7^, ^11^, ^21^, ^23^, ^24^ Regarding the follow‐up period, seven studies with follow‐up ≤3 months^6^, ^17^, ^19^, ^20^, ^21^, ^22^, ^23^ and six studies with 3‐ to 12‐month follow‐up.^7^, ^8^, ^9^, ^11^, ^18^, ^24^ There were seven multi‐center studies^6^, ^7^, ^11^, ^17^, ^18^, ^20^, ^23^ and six single‐center studies.^8^, ^9^, ^19^, ^21^, ^22^, ^24^ Ten studies were judged as high quality (Jadad scale score >3)^6^, ^7^, ^8^, ^9^, ^11^, ^17^, ^18^, ^21^, ^23^, ^24^ and three as low quality (Jadad scale score ≤3).^19^, ^20^, ^22^ The risk of bias in the included studies is shown in Figure 2.

### 3.2 Overall QOL, subgroup, meta‐regression, and sensitivity analyses

**FIGURE 3:**
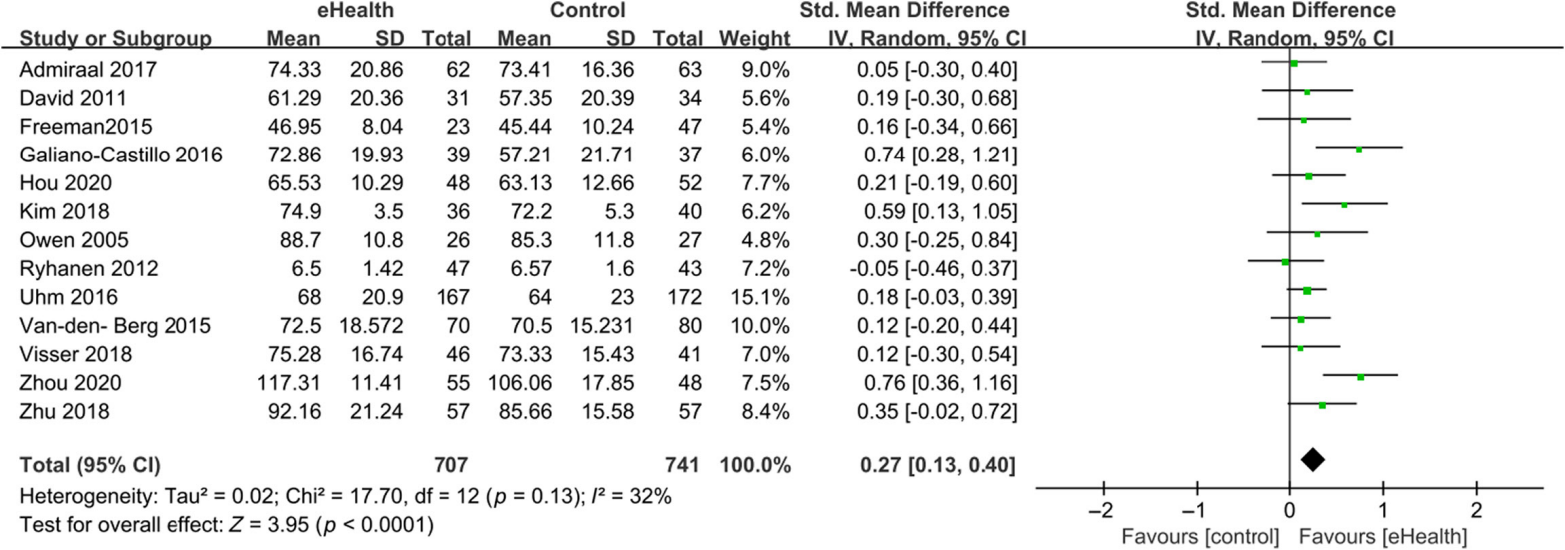
Forest plot for the effect of eHealth on overall quality of life.

Compared with the control group, the eHealth group showed significantly higher QOL scores, with an SMD of 0.27 (95% CI 0.13–0.40, *p* < 0.0001). Heterogeneity testing showed *I* ^2^ = 32%, indicating low heterogeneity (Figure 3).

To explore the potential source of heterogeneity, we conducted subgroup, meta‐regression, and sensitivity analyses. In subgroup analyses shown in Table 3, studies with participants undergoing medical treatment (0.41 [0.07–0.75]), conducted in upper‐middle‐income countries (0.43 [0.12–0.75]), providing mobile‐based eHealth intervention (0.34 [0.15–0.53]) and 3‐ to 12‐month follow‐up duration (0.33 [0.07–0.59]), using breast cancer‐specific QOL scales (0.32 [0.05–0.58]), and published in last 10 years (0.30 [0.15–0.45]) showed numerically more effective influence of eHealth on QOL than those from the counterpart subgroups; but the differences between subgroups were not statistically significant.

**TABLE 3:** Subgroup analyses of QOL.

Subgroup	Stratification	No. of studies	*p* value for heterogeneity	*I* ^2^ (%)	Pooled standardized mean differences
Health status					
	Patients	9	0.14	35	0.26 [0.09, 0.43]
	Survivors	4	0.15	44	0.28 [0.04, 0.52]
Medical treatment					
	Undergoing	4	0.04	64	0.41 [0.07, 0.75]
	Not undergoing	9	0.59	0	0.20 [0.08, 0.32]
Income level					
	High‐income countries	10	0.30	15	0.21 [0.08, 0.34]
	Upper‐middle‐income countries	3	0.14	50	0.43 [0.12, 0.75]
eHealth intervention					
Whether communication with health providers					
	Communication	8	0.31	15	0.27 [0.10, 0.43]
	Non‐communication	5	0.05	32	0.27 [0.13, 0.40]
Whether mobile‐based					
	Mobile‐based	6	0.12	42	0.34 [0.15, 0.53]
	Non‐mobile‐based	7	0.26	22	0.19 [0.01, 0.37]
Follow‐up period					
	≤3 months	7	0.73	0	0.21 [0.07, 0.34]
	3–12 months	6	0.02	62	0.33 [0.07, 0.59]
QOL scales					
	Breast cancer‐specific	5	0.10	49	0.32 [0.05, 0.58]
	Cancer‐specific	6	0.27	21	0.20 [0.04, 0.36]
	General	2	0.21	36	0.38 [−0.04, 0.81]
Study quality					
	High (Jadad scale score >3)	10	0.04	48	0.28 [0.12, 0.44]
	Low (Jadad scale score ≤3)	3	0.93	0	0.21 [−0.08, 0.50]
Publication year					
	Last 10 years	10	0.08	42	0.30 [0.15, 0.45]
	Earlier years	3	0.58	0	0.11 [−0.16, 0.39]

Abbreviations: eHealth, electronic health; QOL, quality of life.

Meta‐regression analyses revealed that the effect sizes did not statistically significantly vary according to participant characteristics, types of eHealth intervention, follow‐up period, assessment scale, study quality, and the publication year (all *p* > 0.30, Table S1). In sensitivity analyses, the pooled estimates were not significantly altered when any one research was omitted in turn, with the SMD ranging from 0.21 (95% CI 0.11–0.32) to 0.29 (0.15–0.43). Therefore, the pooled estimate for QOL is robust in the current study.

### 3.3 Different functional domains of QOL

Our study assessed the effects of eHealth on five functional domains of QOL, including physical, cognitive, social, role, and emotional domains, as measured by the functional subscales of the EORTC QLQ‐C30.

**FIGURE 4:**
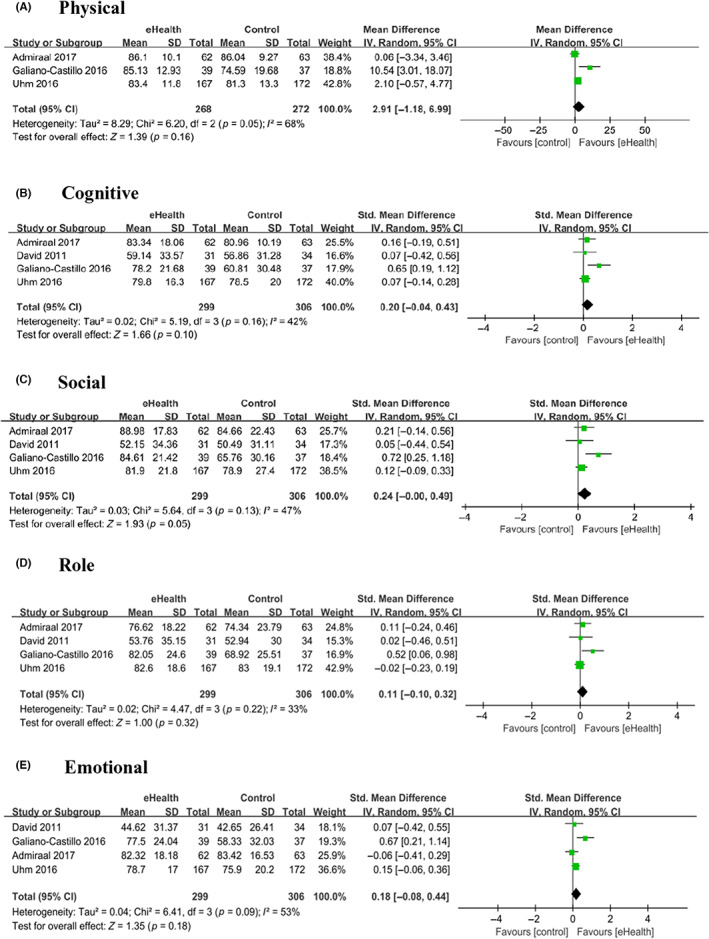
Forest plot for the effect of eHealth on five functional domains. (A) Physical, (B) Cognitive, (C) Social, (D) Role, (E) Emotional.

Physical domain was investigated in three of the studies^9^, ^17^, ^23^ (540 patients), which showed no statistically significant difference between the eHealth and control groups (SMD 2.91, 95% CI −1.18 to 6.99, *p* = 0.16, *I* ^2^ = 68%). The cognitive, social, role, and emotional domains were assessed in four studies involving 605 patients^9^, ^17^, ^19^, ^23^; the results showed no statistically significant difference between the eHealth and control groups (cognitive: SMD 0.20, 95% CI −0.04 to 0.43, *p* = 0.10, *I* ^2^ = 42%; social: SMD 0.24, 95% CI −0.00 to 0.49, *p* = 0.05, *I* ^2^ = 47%; role: SMD 0.11, 95% CI −0.10 to 0.32, *p* = 0.32, *I* ^2^ = 33%; emotional: SMD 0.18, 95% CI −0.08 to 0.44, *p* = 0.18, *I* ^2^ = 53%) (Figure 4).

### 3.4 Anxiety

Four studies involving 231 patients^8^, ^11^, ^19^, ^21^ found no significant difference between the eHealth and control groups (SMD −0.05, 95% CI −0.26 to 0.17, *p* = 0.67). Heterogeneity testing showed *I* ^2^ = 0%, which indicated low heterogeneity (Figure S1).

### 3.5 Depression

Three studies of 255 patients^8^, ^19^, ^21^ found no significant difference between the eHealth and control groups (SMD 0.19, 95% CI −0.05 to 0.44, *p* = 0.12). Heterogeneity testing showed *I* ^2^ = 0%, indicating low heterogeneity (Figure S2).

### 3.6 Publication bias

**FIGURE 5:**
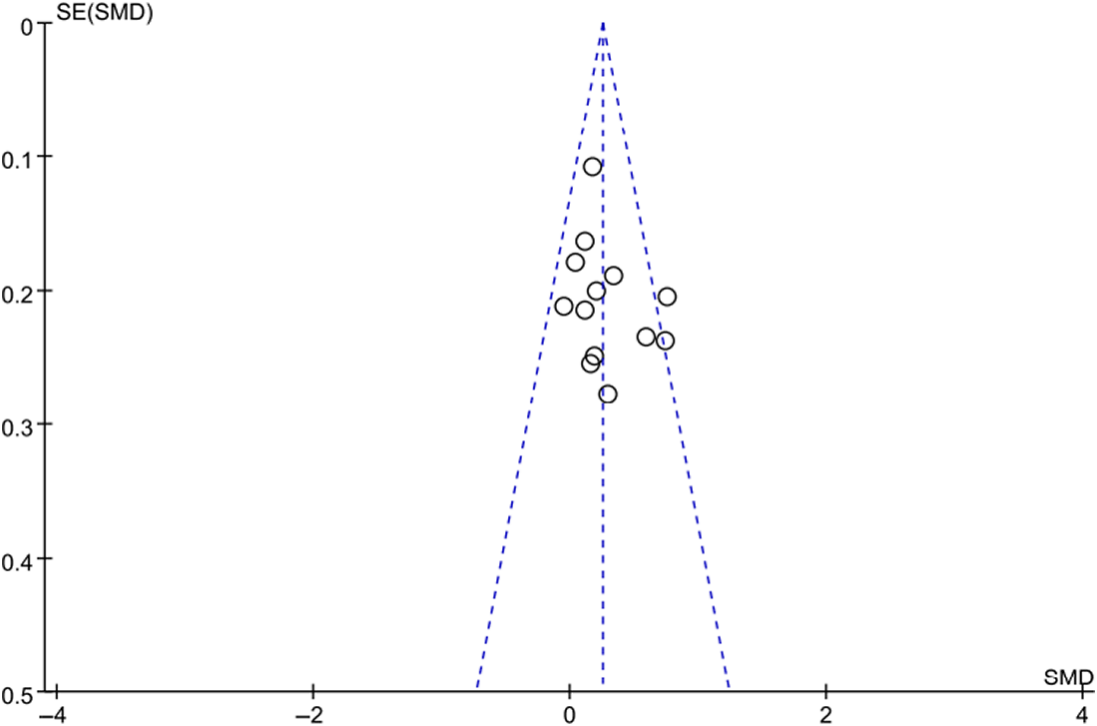
Funnel plot for the studies included in the meta‐analysis. SMD, standard mean difference.

The potential publication bias of QOL studies was performed, as shown in the funnel plot in (Figure 5). The results showed that a publication bias might exist in the research.

## 4 DISCUSSION

To our knowledge, this is the first meta‐analysis to demonstrate the effect of eHealth on overall and specific functional domains of QOL among women with breast cancer. The results revealed that the eHealth group was associated with statistically significant improvement in QOL compared with the usual care group, regardless of participant characteristics, features of the eHealth platforms, and assessment scales. Regarding the functional subscales of EORTC QLQ‐C30, the physical, cognitive, role, and emotional domains of QOL showed no significant group effects. However, the eHealth intervention did experience an improvement tread in these functional domains. In addition, compared with usual care, eHealth did not significantly relieve anxiety and depression in our study. Based on our findings, eHealth is worthy of healthcare use for both breast cancer patients and survivors.

For the intervention effect on QOL, our results are consistent with a previous meta‐analysis which indicated that telehealth intervention was superior to usual care in breast cancer patients for improved QOL.^14^ However, the previous meta‐analysis covered non‐eHealth interventions (e.g., telephone) and did not consider the specific domains of QOL. Our study novelly and specifically evaluated the effect of eHealth on five functional domains of EORTC QLQ‐C30. Since the same scale (EORTC QLQ‐C30) was used for functional evaluation, information bias would be avoided to some extent. The results indicated that eHealth intervention tended to improve these functional domains, although the improvement was not statistically significant. Further confirmation is required for the effect of eHealth on specific health issues.

Our study showed that eHealth did not positively affect anxiety, which was similar to the result of a study about a nurse‐led telephone follow‐up and educational group program after breast cancer treatment.^25^ Although not statistically significant, eHealth tended to relieve depression in our study. The eHealth intervention included in the depression analysis were psychosocial counseling by web‐based e‐mail,^19^ mobile games developed to improve self‐management,^21^ and mobile breast cancer e‐support programs.^26^ A study conducted by Akechi et al.^27^ demonstrated that smartphone psychotherapy reduced the fear of cancer recurrence and depression among breast cancer survivors. Psychotherapy through eHealth may be a promising way to reduce psychological issues.

Considering the different characteristics of participants, intervention, and outcome assessment instruments in the included RCTs, a random effects model was constructed for meta‐analysis, and subgroup analyses were conducted. The use of a random‐effects model measured variability between trials and weighted each study's contribution within the pooled effect. Through subgroup analyses, we found that mobile‐based eHealth intervention, breast‐cancer‐specific QOL scales, and publications within the last 10 years suggested associations with higher effect sizes, and the possible explanations are as follows: (1) mobile‐based eHealth interventions are more portable and utilization efficient than non‐mobile‐based interventions. These results could also indicate the improvement of eHealth intervention patterns in the future; (2) breast cancer‐specific scales are sensitive for the measurement of breast cancer‐specific QOL issue,^28^, ^29^ and (3) studies published within the last 10 years included mobile‐based interventions, while those published earlier only involved non‐mobile‐based interventions. Overall, consistent benefits were observed in both the subgroup and pooled estimates. Therefore, the pooled estimate for QOL is generally robust in the current study.

The results of our study are similar to those of some studies including participants with other cancers.^30^, ^31^, ^32^ One clinical trial showed that tele‐motivational interviewing was an effective and acceptable intervention for overweight participants with cancer (e.g., breast, prostate, uterine, and colorectal cancers, and lymphoma) to improve their QOL.^32^ A meta‐analysis revealed that telehealth interventions were effective and alternative methods for improving QOL among cancer survivors.^30^ More evidence is needed to better introduce eHealth and other relevant telehealth interventions to benefit cancer patients.

To the best of our knowledge, this is the first meta‐analysis to demonstrate the effect of a wide spectrum of eHealth patterns on overall QOL and specific QOL functional domains in women with breast cancer. In addition, this study considers the association between potential covariates (e.g., participant and intervention characteristics) and the effect sizes to verify the stability of the synthesized results. Therefore, our results give a comprehensive perspective on the effect of eHealth on women with breast cancer. This study also has some limitations. First, we only included articles published in English, so we might have missed some pertinent studies. In addition, insufficient data were available to identify the associations between breast cancer stages and the effect of eHealth on QOL. Future meta‐analyses could account for this diversity between studies to avoid ceiling and floor effects.

## 5 CONCLUSIONS

eHealth offers a promising way to improve the overall QOL of women with breast cancer. Implications for clinical practice should be discussed based on subgroup analysis results. Further confirmation is needed for the effect of different eHealth intervention models on specific domains of QOL, which would help improve specific health issues of the target population.

## AUTHOR CONTRIBUTIONS

**Tianmeng Wen:** Data curation (lead); formal analysis (lead); methodology (lead); project administration (lead); resources (lead); software (lead); writing – original draft (lead); writing – review and editing (lead). **Chongxiang Chen:** Data curation (supporting); methodology (supporting); writing – original draft (supporting). **Wenhui Ren:** Supervision (supporting); writing – review and editing (supporting). **Shangying Hu:** Methodology (supporting); writing – review and editing (supporting). **Xuelian Zhao:** Methodology (supporting); writing – review and editing (supporting). **Fanghui Zhao:** Conceptualization (lead); methodology (lead); supervision (lead); writing – review and editing (supporting). **Qinyu Zhao:** Conceptualization (lead); methodology (lead); supervision (lead); writing – review and editing (supporting).

## FUNDING INFORMATION

Cancer Prevention and Control Special Fund from General Electric Company, Cancer Foundation of China.

## CONFLICT OF INTEREST STATEMENT

The authors declare that they have no conflict of interest.

## ETHICS STATEMENT

Not applicable.

## REGISTRATION

The review was not registered.

## Supporting information


Data S1.

Data S2.

## Data Availability

I confirm that the data supporting the findings of this study is from published articles—data are freely available. I confirm that I have included a citation for available data in my references section.
