# Supplementary material for: Effect of electronic health (eHealth) on quality of life in women with breast cancer: A systematic review and meta‐analysis of randomized controlled trials
Source: Cancer Med. 2023 May 18;12(13):14252–63. doi: 10.1002/cam4.6094 (PMC10358200; doi:10.1002/cam4.6094)
Supplement: Supplementary file 1 — Data S1. [file CAM4-12-14252-s001.docx]

**Supplementary MATERIALS List:**

**Supplementary TABLE 1. Multi-covariate meta-regression model**

**Supplementary FIGURE 1. Forest plot for the effect of eHealth on anxiety**

**Supplementary FIGURE 2. Forest plot for the effect of eHealth on depression**

| **Supplementary TABLE 1: Multi-covariate meta-regression model** | | | |
| --- | --- | --- | --- |
| **Covariate** | ***OR*** | **95% CI** | ***p*-value** |
| Health status  (1=patient, 0=survivor) | 0.001 | [1.24e-11, 98584.41] | 0.322 |
| Medical treatment  (1=undergoing, 0=not undergoing) | 155.575 | [3.10e-07, 7.80e+10] | 0.481 |
| Income level  (1=high-income countries,  0=upper-middle-income countries) | 0.000 | [7.02e-19, 5.75e+09] | 0.409 |
| eHealth intervention |  |  |  |
| Communication with health providers  (1=communication, 0=non-communication) | 13.873 | [3.95e-09, 4.88e+10] | 0.729 |
| Mobile-based  (1=mobile-based, 0=non-mobile-based) | 0.413 | [7.61e-08, 2239366] | 0.868 |
| Intervention duration  (1= [＞3 months], 0=[≤3 months]) | 5.846 | [0.000, 2770868] | 0.696 |
| Assessment scale  (1=breast cancer-specific QOL scales,  0=non-breast cancer-specific QOL scales) | 0.000 | [2.95e-16, 1.07e+09] | 0.461 |
| Study quality  (1=high [Jadad scale score >3],  0=low [Jadad scale score≤3]) | 0.534 | [4.17e-11, 6.84e+09] | 0.937 |
| Publication year  (1= Last 10 years, 0= earlier years | 0.098 | [2.79e-13, 3.46e+10] | 0.799 |
| OR: odds ratio; CI: confidence interval; eHealth: electronic health; QOL: quality of life | | | |


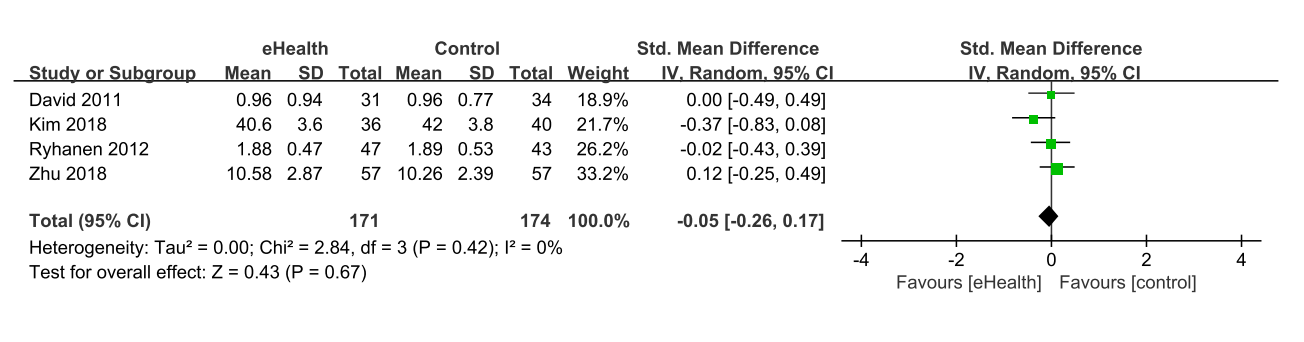


**Supplementary FIGURE 1. Forest plot for the effect of eHealth on anxiety**


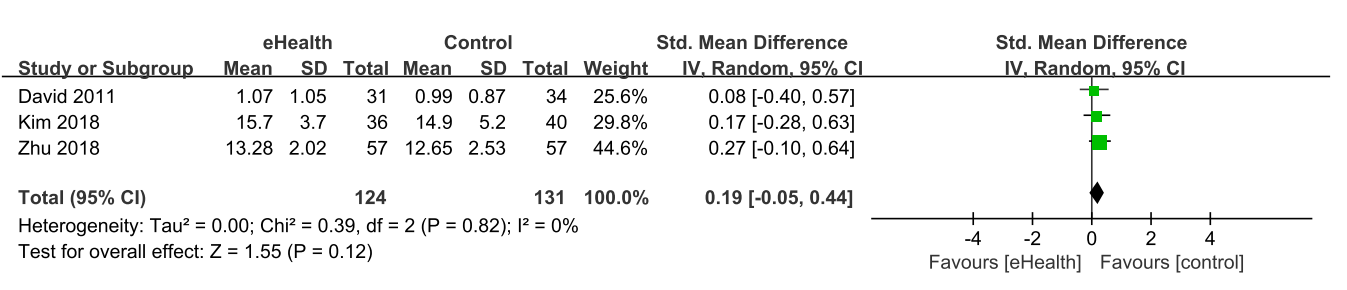


**Supplementary FIGURE 2. Forest plot for the effect of eHealth on depression**
